# The mind-liver connection: Exploring psychological stress and its association with the risk of developing intrahepatic cholestasis of pregnancy

**DOI:** 10.12669/pjms.42.5.13871

**Published:** 2026-05

**Authors:** Shazia Ali, Maryam Khizar Hayat Khan, Sana Nawab, Ghazala Jawwad

**Affiliations:** 1 Shazia Ali, MBBS, MPhil, PhD, PGD, CHPE, CMT, LMPC, Professor, Head of Physiology Department, Islamic International Medical College, Rawalpindi, Pakistan; 2 Maryam Khizar Hayat Khan, BDS, MPhil Physiology, CHPE, Postgraduate Trainee, Islamic International Medical College, Rawalpindi, Pakistan; 3 Sana Nawab, MBBS, MPhil Physiology, CHPE, Islamic International Medical College, Rawalpindi, Pakistan; 4 Ghazala Jawwad, MBBS, MPhil, CHPE, PhD, Associate Professor of Physiology, Bahria University College of Medicine, Islamabad, Pakistan

**Keywords:** Cholestasis, Depression, Pregnancy, Stress

## Abstract

**Objectives:**

To determine the association between intrahepatic cholestasis of pregnancy (ICP) and psychological distress (depression, anxiety, and stress measured using the DASS-21) among Pakistani pregnant women during the second and third trimesters.

**Methodology:**

A prospective observational study was conducted from August 2024 to August 2025 at the Department of Physiology, Islamic International Medical College, jointly with Holy Family Hospital, Rawalpindi and Prime Lab Diagnostics. A total of 140 pregnant women, of ages between 20-40 years, were enrolled after taking informed consent. The DASS-21 questionnaire (Depression, Anxiety and Stress Scale - 21 items) was used in the second trimester and repeated in the third trimester to measure depression, anxiety and stress levels. ICP symptoms such as nocturnal itching were observed throughout pregnancy. Liver function and serum bile acids tests were performed in both second and third trimesters to ascertain the diagnosis. Later, the participants were divided into ICP and non-ICP groups. DASS-21 scores were compared between the two groups.

**Results:**

Depression, anxiety, and stress scores were significantly higher in the ICP group during the third trimester (p < 0.01). Wilcoxon signed-rank tests revealed a prominent rise in depression and stress from the second to third trimester within the ICP group. Mann-Whitney U tests showed depression, anxiety, and stress were higher in the ICP group, particularly in the third trimester (all p < 0.05). Logistic regression identified third-trimester depression (OR: 1.34, p = 0.005) and stress (OR: 1.17, p = 0.026) as significant predictors of ICP. Model fit was acceptable (Hosmer-Lemeshow p = 0.174; AUC = 0.796).

**Conclusion:**

Third-trimester psychological stress, particularly depression and stress symptoms, is associated with ICP. Routine antenatal screening may help identify women at risk for timely intervention.

## INTRODUCTION

Intrahepatic cholestasis of pregnancy is a typical liver related condition that generally manifests during the third trimester and resolves after childbirth. It can impose serious maternal and fetal complications. Women present with severe itching (often worse at night, particularly on the palms and soles), jaundice and frequent nausea. A hallmark of the condition is elevated serum bile acid concentrations (TBA>10μmol/L) and deranged liver function often accompanied by elevated transaminase levels.^1^ ICP has a prevalence of 1% to 27.6% around the world with an incidence of 1.29% in Pakistan.^2^,^3^ Liver disorders during pregnancy are an important clinical concern in Pakistan, and their impact on maternal and fetal outcomes underscores the need for further research, particularly on conditions such as intrahepatic cholestasis of pregnancy (ICP).^4^

Main risk factors for ICP, include advanced maternal age, obesity, personal or family history of ICP, oral contraceptive drugs and women suffering from co-morbidities (hypertension, diabetes).^2^ In ICP, disrupted bile flow from the liver to gallbladder results in compensatory bile acid transport into the bloodstream.^5^ The condition is influenced by genetic, hormonal, immunological, and environmental factors.^6^ The diagnosis of ICP can be made in a pregnant patient in the late second trimester when pruritus without a rash is present, other liver diseases have been excluded and there is elevation of serum bile acids and aminotransferase levels.^7^

Bile acids, derived from cholesterol in the liver, include primary and secondary bile acids. These acids are released into bile, reabsorbed in the ileum, and converted by gut bacteria into additional secondary bile acids. Approximately 95% of these bile acids are recycled back to the liver, while 5% are excreted in feces. Disruption in normal bile flow can result in increased levels of bile acids in the bloodstream.^8^

Known risk factors for ICP do not fully explain why some women develop ICP while others do not. Additionally, cortisol levels correlate with the severity of cholestasis. In cholestasis, higher cortisol levels are associated with more severe disease presentations, suggesting that stress-induced hormonal changes, potentially mediated via activation of the hypothalamic-pituitary-adrenal (HPA) axis might exacerbate cholestatic conditions during pregnancy.^9^ Globally, the prevalence of depression, anxiety and stress symptoms during pregnancy across all trimesters is estimated at 27%, 37%, and 26%, respectively.^10^

Although the prevalence and consequences of ICP such as pruritus, jaundice and adverse perinatal outcomes have been reported in the area, nothing is known about the possible connection between psychological stress and ICP.^3^ This draws attention to a substantial gap in the literature and demonstrates the need for more investigation into the potential effects of psychological stress on ICP. The purpose of this study was to look into any possible connections between psychological stress and the incidence of ICP in expectant mothers. We assessed psychological distress in women who experienced ICP and those who did not during the second and third trimesters using the Depression, Anxiety and Stress Questionnaire. Developing more comprehensive prenatal care techniques that inculcate both physical and mental health assistance during pregnancy can be made easier with an understanding of this connection.

## METHODOLOGY

This prospective observational study was carried out at Holy Family Hospital, Rawalpindi from August 2024 to August 2025.

### Ethical Approval:

It was obtained from the Institutional Review Committee: Islamic International Medical College (Ref No. Riphah/IIMC/IRC/24/1081; Dated: October 28, 2024). Special permission to conduct this research was granted by the Medical Superintendent of Holy Family Hospital. Before being enrolled in the study, each participant gave their written informed consent. The study complied with World Health Organization norms and the ethical principles stated in the Declaration of Helsinki.

Non-probability purposive sampling and predetermined inclusion and exclusion criteria, were used to recruit a cohort of 140 pregnant women between the ages of 20 and 40. Both primigravida and multigravida women between the gestational ages of 13 and 26 weeks were eligible to participate in the study. Women with normal, overweight or obese BMI were included. They had no history of prior liver disease. They also had no symptoms such as jaundice or pruritus. Women under 20 or over 40 were excluded to reduce confounding from age-related physiological and obstetric factors that could independently affect ICP risk. Non-pregnant women were excluded. Those with gestational ages outside 13-26 weeks were also excluded. Any pregnant female with any pre-existing liver disease was not recruited.

A trained doctor interviewed all patients in the OPD in their second trimester and later in the third trimester using the DASS-21 questionnaire. It is a short questionnaire that measures depression, anxiety and stress levels by giving scores for each scale. It contains a total of 21 items, with seven questions in each subscale. Each item is scored from 0 to 3, and the total score for each subscale is multiplied by two to match the original DASS-42 scoring.

Depression scores were classified as: Normal (0-9), Mild (10-13), Moderate (14-20), Severe (21-27), and Extremely Severe (28+). Anxiety scores were: Normal (0-7), Mild (8-9), Moderate (10-14), Severe (15-19), and Extremely Severe (20+). Stress scores were: Normal (0-14), Mild (15-18), Moderate (19-25), Severe (26-33), and Extremely Severe (34+).^11^ These cut-off values were used to identify the level of psychological distress in each participant. The DASS-21 was repeated in the third trimester to observe any changes in depression, anxiety, or stress.

Participants were observed for signs of intrahepatic cholestasis of pregnancy during the course of the study, especially nocturnal itching on the palms and soles of the feet. This is a classic clinical symptom of the disease. In order to support the diagnosis, the existence of these symptoms was noted during each interview and assessed along with laboratory testing.

Liver function tests and serum bile acids were measured in both second and third trimesters. ICP was diagnosed when serum total bile acids exceeded 10 μmol/L in the presence of characteristic pruritus. Blood samples were taken between 8:00 and 10:00 a.m. to maintain consistency and reduce diurnal variation, particularly of bile acids. About 5 mL of venous blood was drawn from the medial cubital vein into a serum separator tube and allowed to clot. After that, it was centrifuged. To maintain sample quality the cold chain was maintained by keeping serum samples in an ice box during handling and transport until they were stored at –80°C. Serum alanine aminotransferase, aspartate aminotransferase, alkaline phosphatase and total bile acids were all included in the biochemical study. Increased bile acid levels, abnormal liver function tests and clinical symptoms were used to diagnose ICP.

Participants were categorized into two groups after assessment. One group included women diagnosed with ICP. The other group consisted of women without ICP. To explore whether psychological stress was linked to ICP, we compared their DASS-21 scores. Data were analysed using SPSS version 27 and Julius AI (Version 1.0.45). Continuous variables were summarized as medians and interquartile ranges, while categorical variables were reported as frequencies. Differences within groups were examined using Wilcoxon signed-rank tests, while differences between groups were evaluated using Mann-Whitney U tests. Data were not normally distributed, making non-parametric methods more appropriate for comparing medians and paired observations. Correlations and Variance Inflation Factor (VIF) evaluated multicollinearity. Logistic regression identified predictors of ICP, with p < 0.05 as significant. Model fit was assessed using the Hosmer-Lemeshow test, and predictive accuracy by AUC.

## RESULTS

A total of 140 pregnant women were analyzed, including 70 with Intrahepatic Cholestasis of Pregnancy (ICP) and 70 without (non-ICP). The median age was consistent across both groups, with 30 years in the ICP group and 29 years in the non-ICP group. Median BMI was slightly higher in the ICP group (28.2 kg/m²) than in the non-ICP group (26.5 kg/m²). The majority of participants were multiparous in both groups. Demographic data, including age and BMI stratified by disease and parity groups, are presented in Table-I.

**Table-I:** Descriptive Statistics of Age and BMI by Disease and Parity Groups.

Category	N	Age (Median, IQR)	BMI (Median, IQR)
ICP	70	30 (27-34)	28.2 (25.3-30.6)
Non-ICP	70	29 (26-33)	26.5 (24.1-29.8)
Primigravida	54	27 (24-30)	25.4 (22.3-28.1)
Multigravida	86	31 (28-34)	27.9 (25.1-30.3)

ICP = Intrahepatic Cholestasis of Pregnancy.

Psychological distress, measured using the DASS-21 scale, was consistently higher in women who developed ICP compared to those who did not. In the second trimester (T2, 13-26 weeks), median scores in the ICP group were 19 (IQR 16.25-22) for depression, 18 (IQR 14–20) for anxiety, and 18 (IQR 15-21) for stress, compared to 17 (IQR 14.25-20), 14 (IQR 12-18), and 16 (IQR 12-20) in the non-ICP group. By the third trimester (T3, 27-42 weeks), scores increased in both groups, with the ICP group reaching 24 (IQR 21.25-24) for depression, 22 (IQR 17-24.75) for anxiety, and 24 (IQR 19-27) for stress, while the non-ICP group scored 20 (IQR 17-23.75), 18 (IQR 15-20), and 18.5 (IQR 17-22), respectively.

Within-group changes from T2 to T3 were statistically significant for both ICP and non-ICP groups, as assessed by the Wilcoxon signed-rank test (all p < 0.001), indicating a significant increase in depression, anxiety, and stress as pregnancy progressed. Notably, the magnitude of increase was greater in the ICP group for depression and stress, while anxiety showed a modest increase in both groups, suggesting that women who developed ICP experienced a more pronounced worsening of psychological distress.

Between-group comparisons using the Mann-Whitney U test showed that at T2, depression (p = 0.011), anxiety (p = 0.00033), and stress (p = 0.021) were significantly higher in the ICP group. At T3, differences were even more pronounced, with depression (p < 0.001), anxiety (p < 0.001), and stress (p < 0.001) all significantly higher in the ICP group. These findings indicate that psychological distress was consistently greater in women who developed ICP and worsened from the second to third trimester, with the largest increases observed in the third trimester.

Bowker’s test indicated significant changes in the frequency of depression, anxiety, and stress severity from the second (T2) to third trimester (T3) in both ICP (diseased) and non-ICP (non-diseased) groups (p < 0.001). In the ICP group, severe depression increased from 31.4% at T2 to 71.4% at T3, extremely severe depression reached 10%, extremely severe anxiety rose from 35.7% to 64.3%, and severe stress grew from 2.9% to 42.9%. In the non-ICP group, severe depression increased from 18.6% to 42.9%, extremely severe anxiety from 8.6% to 31.4%, and severe stress from 2.9% to 7.1%. These results indicate that psychological distress worsened over time in both groups, with a more pronounced increase in severity among women who developed ICP. The frequency of severity levels for stress, anxiety, and depression from the second to the third trimester in both the ICP (diseased) and non-ICP (non-diseased) groups is shown in Table-II.

**Table-II:** Frequency of Severity Levels from Second to Third Trimester in ICP and Non-ICP Groups (N = 140).

Symptom	Severity Level	ICP T2 (%)	ICP T3 (%)	Non-ICP T2 (%)	Non-ICP T3 (%)
Depression	Normal	0	0	4.3	0
	Mild	60	17.1	64.3	45.7
	Moderate	8.6	1.4	11.4	7.1
	Severe	31.4	71.4	18.6	42.9
	Extremely Severe	0	10	1.4	4.3
Anxiety	Normal	4.3	1.4	7.1	1.4
	Mild	24.3	14.3	40	17.1
	Moderate	2.9	1.4	5.7	2.9
	Severe	32.9	18.6	38.6	47.1
	Extremely Severe	35.7	64.3	8.6	31.4
Stress	Normal	22.9	4.3	40	11.4
	Mild	35.7	35.7	27.1	38.6
	Moderate	38.6	14.3	28.6	38.6
	Severe	2.9	42.9	2.9	7.1
	Extremely Severe	0	2.9	1.4	4.3

ICP = Intrahepatic Cholestasis of Pregnancy; T2 = Second Trimester; T3 = Third Trimester.

Correlation analysis revealed associations between repeated measures of the same psychological constructs, particularly between second- and third-trimester depression (r = 0.80) and anxiety scores (r = 0.88–0.90), reflecting temporal stability of these variables. Variance Inflation Factor (VIF) values for these predictors ranged from 5.2 to 5.9, indicating moderate correlation across trimesters. While classical guidelines suggest VIF > 5 may indicate potential multicollinearity, recent methodological work emphasizes that such thresholds are context-dependent and do not automatically preclude inclusion of correlated predictors in regression models.^12^ In this study, both second and third trimester scores were retained in multivariable models to explore temporal contributions of psychological factors to the outcome, with all results interpreted in light of these correlations.

T3 depression (p = 0.005), T3 stress (p = 0.026) and T2 depression (p = 0.031) were found to be significant predictors of disease status using multivariable logistic regression. Particularly, the odds of ICP increased by 34% (OR: 1.34) for every unit rise in the T3 depression score and by 17% (OR: 1.17) for every unit increase in the T3 stress level. It’s interesting to note that decreased probabilities of illness were associated with higher T2 depression ratings (OR: 0.79). Parity age, BMI and anxiety ratings were among the other variables that did not reach statistical significance. The findings of the logistic regression analysis are shown in Table-III.

**Table-III:** Logistic Regression Model Predicting ICP (n = 140).

Predictor	OR (95% CI)	p-value
Parity	0.75 (0.28-1.93)	0.55
Age	1.06 (0.98-1.16)	0.17
BMI	1.05 (0.93-1.21)	0.45
T2 Depression	0.79 (0.63-0.97)	0.031 *
T2 Anxiety	1.04 (0.86-1.26)	0.69
T2 Stress	0.90 (0.78-1.04)	0.17
T3 Depression	1.34 (1.10-1.66)	0.005 *
T3 Anxiety	1.04 (0.86-1.25)	0.70
T3 Stress	1.17 (1.02-1.35)	0.026 *

* p ≤ 0.05 (statistically significant).

Model evaluation indicated good performance. Hosmer-Lemeshow test with p = 0.174, indicated that the model fit the data appropriately. The ROC curve showed an AUC of 0.796, reflecting good discrimination between ICP and non-ICP cases. Using Youden’s index, the optimal predicted probability cut-off was 0.4, which yielded a sensitivity of 82.9% and specificity of 70.0% for detecting ICP. These metrics provide a clinically interpretable threshold for identifying high-risk pregnancies based on trimester-specific psychological symptoms. Overall, the results indicate that changes in depression and stress across pregnancy, particularly in the third trimester, are strongly associated with ICP, while demographic factors added little to the model.

## DISCUSSION

Increased psychological stress as determined by the DASS 21 scale was found to be significantly associated with the development of intrahepatic cholestasis during pregnancy, according to the current study. Third-trimester stress and depression scores were considerably higher in ICP participants than in non-ICP participants, suggesting increased psychological suffering. Both the variables were found to be independently associated with ICP in logistic regression analysis. These findings add new insights to existing research exploring psychosomatic influences on pregnancy-related hepatobiliary disorders.^13^ Previous studies have mostly focused on the impact of ICP on psychological well-being, with limited exploration of the reverse. Yeşil et al., reported that women with ICP experienced greater emotional distress and poorer quality of life scores compared to normative data.^14^

However, unlike the current study, their study was cross-sectional and did not assess psychological status prior to ICP diagnosis. Hualin et al., in a follow-up study, found significantly higher rates of antenatal and postnatal depressive symptoms among those diagnosed with intrahepatic cholestasis of pregnancy (ICP) compared to healthy controls, as measured by validated scales, and treatment with ursodeoxycholic acid did not alleviate depressive symptoms.^15^ Our findings are novel in that we used a validated scale (DASS-21) longitudinally and reveal that psychological distress in the third trimester precedes and predicts ICP onset.

The DASS-21 has been used in diverse maternal health studies and shows good psychometric performance in pregnancy; in Pakistan, the Urdu version of the DASS-21 was employed to assess prenatal depression, anxiety, and stress among pregnant women in Karachi, demonstrating its applicability for evaluating psychological distress and its relation to birth outcomes in obstetric population.^16^ A more recent study by Motta et al., applied the DASS21 in a prospective cohort of 109 IVF-conceived pregnancies at 12-, 24-, and 34-weeks’ gestation. While overall scores remained stable, the tool demonstrated strong feasibility and sensitivity by detecting elevated stress and anxiety in subgroups.^11^ These findings support our use of the DASS21 questionnaire for screening stress during pregnancy.

Our study’s prospective design, which permits temporal causality, is one of its main advantages. Robust analytical methods, such as logistic regression, evaluated using model fit indices like the Hosmer-Lemeshow test, increase internal validity. Causal inference has been limited by the majority of previous investigations’ reliance on post-diagnosis psychological evaluation.^15^ Our longitudinal method improves interpretation and raises the possibility that psychological stress could be a controllable risk factor for the disease.

Stress can biologically contribute to the development of ICP through various pathways. When the hypothalamic-pituitary-adrenal axis is activated under stress, levels of glucocorticoids and catecholamines rise, which may in turn disrupt bile acid metabolism and the function of hepatic transporters.^17^,^18^ Along with that, psychological stress is known to trigger oxidative stress and the release of inflammatory cytokines, processes that are associated with liver dysfunction and disorders such as ICP.^19^ These mechanisms provide a valid explanation for how mental distress may impact hepatic health during pregnancy.

Our results highlight the need of incorporating psychological evaluations into standard prenatal care. Women who are at risk of getting ICP can be identified by early detection of stress and depression, particularly during the third trimester. Prenatal stress can be reduced with the use of techniques like mindfulness, cognitive-behavioral therapy, and stress management courses.^20^,^21^ This study provides preliminary evidence suggesting an association between psychological distress in late pregnancy and ICP. Future interventional studies are warranted to evaluate whether targeting psychological distress could have a beneficial effect on ICP outcomes.

### Limitations:

Despite its strengths, this study also has several limitations. Since all participants were recruited from one tertiary care center, the findings may not be generalizable to other populations or settings. Some psychological subgroups, such as women with extremely severe depression, had small sample sizes, which reduces statistical power for these categories.

## CONCLUSION

This prospective study concludes that psychological distress during the third trimester is strongly correlated with intrahepatic cholestasis of pregnancy. Stress and depression as measured by the validated DASS-21 scale were independent predictors of ICP. These findings highlight the advantages of using mental health treatments to lessen pregnancy related liver issues and suggest that routine psychological testing is essential during pregnancy. This study is the first in Pakistan to evaluate psychological distress in women with Intrahepatic Cholestasis of Pregnancy using the DASS-21 scale.

### Recommendations:

Future studies should also include larger, multicenter cohorts, consider structured psychiatric interviews, utilize probability sampling techniques and explore interventions aimed at reducing psychological distress in pregnancy to further validate and extend these findings.

### Authors’ Contribution:

**SA:** Conceived and designed the study, provided overall supervision, guided methodology and interpretation, and critically reviewed and approved the final manuscript.

**MKHK:** Performed data collection, data entry, statistical analysis, interpretation of results, manuscript drafting and editing, and is responsible for the integrity of the research.

**SN:** Assisted in data collection, literature review, and drafting and revising manuscript sections.

**GJ:** Reviewed the study design, provided expert academic input, and critically reviewed the manuscript for intellectual content.

## References

[1] Axelsen SM, Schmidt MC, Kampmann U, Grønbæk H, Fuglsang J (2024). The effect of twin pregnancy in intrahepatic cholestasis of pregnancy: a case control study. Acta Obstet Gynecol Scand.

[2] Niemyjska-Dmoch W, Kosiński P, Węgrzyn P, Luterek K, Jezela-Stanek A (2023). Intrahepatic cholestasis of pregnancy and theory of inheritance of the disease: literature review. J Matern Fetal Neonatal Med.

[3] Anwar R, Razzaq K, Shahid A, Afsheen A, Tariq A, Saleem I (2021). Evaluation of risk factors for intrahepatic cholestasis of pregnancy in patients presenting to a tertiary care centre. Pak Armed Forces Med J.

[4] Butt N, Ali S, Yasmeen H, Mumtaz K (2024). Outcomes of liver diseases in pregnant females: A study from a tertiary care medical center in Pakistan. Pak J Med Sci.

[5] Yang Z, Yao M, Zhang C, Hu X, Zhong Y, Xu X (2022). Application of metabolomics in intrahepatic cholestasis of pregnancy: a systematic review. Eur J Med Res.

[6] Giouleka S, Zafeirati C, Siargkas A, Michos G, Stavros S, Potiris A, Mamopoulos A, Kalogiannidis I, Dagklis T, Tsakiridis I (2025). Intrahepatic Cholestasis of Pregnancy: A Comparative Review of Guidelines. Obstet Gynecol Surv.

[7] Hague WM, Briley A, Callaway L, Nitert MD, Gehlert J, Graham D (2023). Intrahepatic cholestasis of pregnancy - diagnosis and management: a consensus statement of the Society of Obstetric Medicine of Australia and New Zealand (SOMANZ):executive summary. Aust N Z J Obstet Gynaecol.

[8] Li X, Wang H, Li Y, Huang M, Li X, Yu Z (2025). Pathologic and therapeutic insights into cholestatic liver disease-associated depressive disorder. Acta Materia Medica.

[9] Theiler-Schwetz V, Schlager H, Obermayer-Pietsch B, Stojakovic T, Fauler G, Fickert P (2021). Hypercortisolism in patients with cholestasis is associated with disease severity. BMC Gastroenterol.

[10] Aziz HA, Yahya HD, Ang WW, Lau Y (2025). Global prevalence of depression, anxiety, and stress symptoms in different trimesters of pregnancy: a meta-analysis and meta-regression. J Psychiatr Res.

[11] Motta CC, Duek JD, Soares LO, Torelli FR, Chehin MB, Lorenzon AR (2022). Emotional aspects assessed by the DASS-21 scale in pregnant women conceived by in vitro fertilization during the COVID-19 pandemic. Fertil Steril.

[12] Alharthi MF (2025). Novel Data-Driven Shrinkage Ridge Parameters for Handling Multicollinearity in Regression Models with Environmental and Chemical Data Applications. Axioms.

[13] Kerachi AJ, Shahlaee MA, Habibi P, Ebrahimi ND, Ala M, Sadeghi A (2025). Global and regional incidence of intrahepatic cholestasis of pregnancy: a systematic review and meta-analysis. BMC Med.

[14] Yeşil Y, Gündüz Ü, Dönmez A, Paşa S (2024). Evaluation of Prenatal Comfort, Sleep, and Quality of Life in Pregnant Women with Cholestasis: A Cross-Sectional Study. Healthcare (Basel).

[15] Hualin X, Yupin X, Guoqiang Z, Xukun F, Hongmei L (2023). Intrahepatic cholestasis of pregnancy worsening perinatal depressive tendency: a follow-up study from the second trimester to the sixth week postpartum. Heliyon.

[16] Nazir F, Rizvi SA, Munir T (2025). Impact of prenatal depression, anxiety and stress on low birth weight: a retrospective case-control study in Karachi, Pakistan. BMJ Public Health.

[17] Theiler-Schwetz V, Schlager H, Obermayer-Pietsch B (2021). Hypercortisolism in patients with cholestasis is associated with disease severity. BMC Gastroenterol.

[18] Richardson AE, VanderKaayTomasulo MM (2022). Stress-induced HPA activation in virtual navigation and spatial attention performance. BMC Neurosci.

[19] Wang M, Chen L, Li J, You Y, Qian Z, Liu J (2024). An omics review and perspective of researches on intrahepatic cholestasis of pregnancy. Front Endocrinol.

[20] Pan WL, Lin LC, Kuo LY, Chiu MJ, Ling PY (2023). Effects of a prenatal mindfulness program on longitudinal changes in stress, anxiety, depression, and mother-infant bonding of women with a tendency to perinatal mood and anxiety disorder: a randomized controlled trial. BMC Pregnancy Childbirth.

[21] Gökbulut N, Cengizhan SÖ, Akça EI, Ceran E (2024). The effects of a mindfulness-based stress reduction program and deep relaxation exercises on pregnancy-related anxiety levels: a randomized controlled trial. Int J NursPract.

